# The role of ubiquitin-specific peptidases in cancer progression

**DOI:** 10.1186/s12929-019-0522-0

**Published:** 2019-05-27

**Authors:** Ming-Jer Young, Kai-Cheng Hsu, Tony Eight Lin, Wen-Chang Chang, Jan-Jong Hung

**Affiliations:** 1 Department of Biotechnology and Bioindustry Sciences, National Cheng Kung University, Tainan, 701 Taiwan; 2 Graduate Institute of Cancer Biology and Drug Discovery, College of Medical Science and Technology, Taipei Medical University, Taipei, Taiwan; 3 Ph.D. Program for Cancer Molecular Biology and Drug Discovery, College of Medical Science and Technology, Taipei Medical University, Taipei, Taiwan; 4 Biomedical Commercialization Center, Taipei Medical University, Taipei, Taiwan; 5 The Ph.D. Program for Neural Regenerative Medicine, Taipei Medical University, Taipei, Taiwan; 6 Graduate Institute of Medical Sciences, College of Medicine, Taipei Medical University, Taipei, Taiwan

**Keywords:** Ubiquitination, Deubiquitinases, Ubiquitin-specific peptidases, Cancer

## Abstract

Protein ubiquitination is an important mechanism for regulating the activity and levels of proteins under physiological conditions. Loss of regulation by protein ubiquitination leads to various diseases, such as cancer. Two types of enzymes, namely, E1/E2/E3 ligases and deubiquitinases, are responsible for controlling protein ubiquitination. The ubiquitin-specific peptidases (USPs) are the main members of the deubiquitinase family. Many studies have addressed the roles of USPs in various diseases. An increasing number of studies have indicated that USPs are critical for cancer progression, and some USPs have been used as targets to develop inhibitors for cancer prevention. Herein we collect and organize most of the recent studies on the roles of USPs in cancer progression and discuss the development of USP inhibitors for cancer therapy in the future.

## Background

After translation, most proteins can undergo various modifications, namely, phosphorylation, acetylation, methylation, sumoylation, glycosylation and ubiquitination, to modulate their activity. Posttranslational modification (PTM) of proteins is an important component of all physiological processes that functions by regulating various pathways, including protein degradation, DNA repair activity, gene regulation and signal transduction [1]. Evolutionarily higher plants and animals have more complex PTMs, indicating that the PTM process is beneficial to supporting the progression of life [2]. Ubiquitin is a small 76-amino-acids protein that can be conjugated to specific target proteins in various forms, namely, polyubiquitination and monoubiquitination. Three types of enzymes, namely, ubiquitin-activating enzymes (E1s), ubiquitin-conjugating enzymes (E2s), and ubiquitin ligases (E3s), are responsible for adding the ubiquitin into target proteins [3]. Seven lysine residues in ubiquitin provide different types of linkages, including monoubiquitination, polyubiquitination and branched ubiquitination, to regulate the different functions of target proteins [4]. Protein monoubiquitination affects DNA repair activity, gene regulation, molecule trafficking and endocytosis [5]. Lys48-linked protein polyubiquitination affects protein degradation in a 26S proteasome-dependent manner. Lys63-linked protein polyubiquitination is involved in DNA repair activity, signal transduction, trafficking and endocytosis [6]. Branched ubiquitination of proteins, such as in the APC/C complex, is also associated with 26S proteasome-dependent degradation [4]. All types of ubiquitination as a protein modification are crucial to maintaining normal physiological conditions [7]. Dysregulation of protein ubiquitination leads to many diseases, including degenerative diseases and cancer [8, 9].

Deubiquitinases (DUBs) are a group of enzymes that are able to remove ubiquitin from ubiquitinated proteins, including monoubiquitinated, polyubiquitinated and branch polyubiquitinated proteins, leading to the regulation of the stability or activity of the target proteins [10, 11]. More than one hundred deubiquitinases that regulate all protein deubiquitination have been identified in humans. DUB members can be divided into five types: ubiquitin-specific proteases (USPs), ovarian tumor proteases (OTUs), ubiquitin C-terminal hydrolases (UCHs), Machado-Joseph disease protein domain proteases (MJDs) and JAMM motif proteases [12, 13]. USPs, OTUs, UCHs and MJDs are cysteine-dependent proteases [14, 15]. The JAMM motif is a metal-dependent protease [14, 15]. Most of these enzymes exert their functions by reversing the polyubiquitination or monoubiquitination of target proteins. An increasing number of studies have indicated that dysregulation of the DUB causes malfunction of the ubiquitin system, which can either increase the effects of oncogenes or decrease the tumor suppressor gene activity. Herein we collected and organized all recent studies that address the roles of each USP in cancer progression.

### The roles of USPs in tumorigenesis

Many studies indicate that USPs regulate tumor formation by modulating the proliferation and death of cancer cells. All USPs and their substrates are shown in Table 1.

**Table 1:** Human ubiquitin specific proteases (USPs) and their reported functions in the cancer progression

Gene symbol	Cellular location	Substrate	Function and remarks in cancer	Inhibitor	References
USP1	N	FANCD2 PCNA	DNA repair; Oncogene	Pimozide^b^, ML323, GW7647, C527, 6-Amino-pyrimidines, SJB2-043, SJB3-019A, PR619	[92, 110–114]
USP2	C, N	Fatty acid synthase, cyclin D1, MDM2 and 4	Fas/p53, NF-κB, c-Myc; Oncogene	NSC632839, AM146, RA-9, RA-14, 2-cyano-pyrimidines and -triazines^b^, ML364, PR619	[18, 31, 44, 114–122]
USP3	N	H2A, H2B	DDR, Oncogene		[123–125]
USP4	C, N	TRAF2, TRAF6	TGFβ, NFκB, Wnt, p53; Oncogene	Vialinin A, PR619	[81, 114, 126–128]
USP5	L, V, C^a^		p53, DDR, Oncogene	G9, Vialinin A, WP1130, EOAI3402143, AM146, RA-9, RA-14, PR619	[49, 93, 106, 114, 118, 127, 129–132]
USP6	Golgi, C		NFκB activation; Oncogene or Suppressor		[133]
USP7	N, C, PML body	HDM2, p53, H2B, TP53, MDM2 & 4, FOXO4, PTEN	Oncogene	P5091, Cpd14, P22077, HBX41108, HBX 19818, HBX 28258, NSC632839, WO2013030218, P0050429, W02013030218, PR619	[114, 117, 121, 134–146]
USP8	C, N	NRDP1, RNF128, STAM2	Oncogene	HBX90397, HBX41108, AM146, RA-9, RA-14, Ethyloxyimino-9H-indeno[1,2-b] pyrazine-2,3-dicarbonitrile, PR619	[95, 114, 118, 147–150]
USP9X	C, E, L, V	β-catenin, epsins, AF-6, SMAD2	TGFβ, Mcl-1, ERG, AGS-3, ITCH, Wnt, Notch; Oncogene or suppressor	G9, WP1130, PR619	[106, 107, 114, 130–132, 151–154]
USP9Y	C		Spematogenesis		[155]
USP10	C, N	TP53, SNX3, CFTR	c-Myc, p53; Oncogene or suppressor	P22077, HBX19818, Spautin-1, PR619	[32, 56, 114, 156–158]
USP11	N, C	BRCA2, NFκBIA	DDR, NFκB; Oncogene	Mitoxantrone^b^	[70, 104, 159–161]
USP12		Androgen receptor	Oncogene	GW7647	[92, 162–164]
USP13	L, V, C, N^a^	MCL1, BECN1, USP10		Spautin-1	[157, 165–167]
USP14	C, PM		Wnt; Oncogene	VLX1570^b^, IU1, WP1130, b-AP15, AC17, Auranofin^b^, Tricyclic heterocyclics, Azepan-4-ones, PR619	[106, 114, 132, 168–175]
USP15	C, N	RBX1, SMAD1, 2, 3 & 7	NFκB, Wnt; Oncogene	PR619	[114, 176–179]
USP16	N	H2A	Chromosome condensation; Oncogene	PR619	[114, 180–183]
USP17		SUDS3	Oncogene		[184–186]
USP18	C, N	TAK1, TAB1, PTEN	JAK-STAT, NFκB; Oncogene		[187, 188]
USP19	ER	RNF123	ERAD	PR619	[114, 189–191]
USP20	C, N,	DIO2, ADRB2, TRAF6, Tax	Thyroid hormone, hypoxia, NFκB; Oncogene	PR619	[114, 192, 193]
USP21	C, N	H2A, RIPK1, DDX58, GATA3, IL33	NFκB, NEDD8; Oncogene		[72, 194–198]
USP22	N	H2A	c-Myc; Oncogene	PR619	[114, 199–202]
USP24	C	TP53, DDB2, MCL1, Bax, p300, E2F4, securin, βTrCP	Cell growth repressor; Metastasis promoter; Overexpression in M2 macrophages	G9, PR619	[23, 57, 75, 106, 114, 130, 131, 203]
USP25	C, N	DDX58	ERAD; Oncogene		[204–206]
USP26	N (testis)	AR	Spermatogenesis		[207–209]
USP27X		BCL2L11	tumor suppressor		[58, 210]
USP28	N		CLSPN, c-MYC; Oncogene or suppressor	PR619	[114, 211, 212]
USP29	N^a^		p53 pathway; Oncogene		[213, 214]
USP30	M	MFN1, MFN2, DRP1, Parkin	Hepatocarcinogenesis		[215–217]
USP31	N, C		Inhibition of NFκB		[218]
USP32	PM, Golgi		Oncogene		[219]
USP33	C, N, centrosome	HIF1-α DIO2, ADRB2, CCP110, ARRB	Tumor suppressor		[192, 220–223]
USP34	C, N, PM, Extracellular	AXIN1, AXIN2,	Activation of Wnt; Inhibition of EMT and cancer stemness		[102, 224]
USP35	N^a^	ABIN-2, Aurora B	Tumor suppressor through inactivating NFκB		[225, 226]
USP36	N	c-Myc	Oncogene		
USP37	N,	c-Myc	Increase in DNA damage repair; Oncogene		
USP38	C, N, GA^a^				
USP39	N		Oncogene		
USP40	C, N, PM				
USP41	N^a^				
USP42	N^a^	TP53	p53; Oncogene		[53, 227, 228]
USP43	N^a^	H2BK120	Tumor suppressor		
USP44	N	CDC20, EZH2	Oncogene		[229]
USP45	C, N				
USP46	L, V^a^		Oncogene	Pimozide^b^	[113]
USP47	C	POLB	Oncogene	P5091, Cpd14, P22077, PR619	[114, 136, 137, 139, 230]
USP48	C, N	Gli1	Oncogene	PR619	[114]
USP49	N	H2B	Tumor suppressor		[231]
USP50	N^a^		G2/M checkpoint		[232]
USP51	N^a^				
PAN2	C, N				
USP53	Golgi, N^a^				
USP54	M^a^		Oncogene		
USPL1	N, Cajal body				
DUB3		H2AX	G2/M checkpoint; Cancer associated		[233, 234]
CYLD	C, N, PM	TRAF2/6, NEMO, TRPA1, Tak1, Lck, Bcl3, Dvl, DDX58, K63polyUb-RIPK1, K63polyUβ-IKBKG	NFκB and JNK-STAT; Familial tumor suppressor		[68, 235–239]

The roles of USPs in the cancer progression. ^a^predicted; C: Cytoplasm; N: Nucleus; L: Lysosome; V: Vacuole; ER: Endoplasmic reticulum; M: Mitochondria; E: Endosome; ^b^Clinic trial on going (https://clinicaltrials.gov/ct2/home)

#### USPs are involved in cell cycle progression

Protein ubiquitination is important for the regulation of cell cycle progression. Ubiquitinases, namely, E1/E2/E3, are well studied. Recently, several deubiquitinases have been reported to be involved in cell cycle progression. USP2 and USP22 can stabilize cyclin D1 to promote cell cycle progression [16, 17]. A recent study also revealed that a small molecule, ML364, can inhibit USP2 to promote degradation, leading to cell cycle arrest [18]. USP7 has been reported to promote the growth of non-small cell lung cancer cells by stabilizing Ki-67 protein [19]. However, metformin can inhibit esophageal cancer proliferation through the upregulation of USP7, suggesting that USP7 has different effects on tumorigenesis in the different cancer types [20–22]. USP24 stabilizes securin to block the cell cycle progression from metaphase to anaphase, leading to cell cycle arrest [23]. According to previous studies, APCC, as an E3-ligase in mitosis, regulates many factors, including securin, to promote cell cycle progression [24]. In addition to E3-ligases, deubiquitinases, such as USP24, may also be important for cell cycle progression [23]. More evidence is needed to support the hypothesis that downregulation of USP24 in mitosis is induced by APCC. USP37 also regulates the stability of oncogenic fusion protein PLZF/RARA [25]. USP37 links REST to the control of p27 stability and cell proliferation [26]. USP44 promotes prostate cancer tumorigenesis by stabilizing EZH2 [27]. USP44 also induces DNA aneuploidy in gastric cancer, which may induce cell cycle arrest and apoptosis [28]. Therefore, USP44 is a tumor suppressor against chromosome missegregation [29]. In addition, USP44 function as an integral component of N-CoR to regulate gene expression [30].

#### USPs-stabilized c-Myc promotes cancer formation

c-Myc is an oncogene that regulates gene expression and cell cycle progression. USP2 is reported to be involved in activating the c-Myc pathway to regulate prostate cancer formation [31]. USP10 can stabilize c-Myc expression [32]. USP22 promotes the proliferation, migration and invasion abilities of glioma, gastric cancer and colorectal cancer [33–35]. In addition, USP22 stabilizes c-Myc to promote breast cancer progression [36]. USP28 contributes to the proliferation and metastasis of gastric cancer [37]. The loss of USP28 enhances the radiosensitivity of esophageal cancer cells via the c-Myc pathway [38]. USP36 stabilizes c-Myc to promote ovarian cancer formation [39]. USP37 directly stabilizes c-Myc in lung cancer [40]. All the studies reveal that USPs are important in regulating c-Myc stability during tumorigenesis.

#### USPs regulate apoptosis-related factors

p53 is a tumor suppressor, and p53 degradation or mutations are critical factors in cancer formation [41]. Several E3 ligases, such as MDM2, have been well studied [42]. Recent studies have also indicated that several deubiquitinases are involved in the regulation of p53 degradation [43]. USP2 and USP7 stabilize MDM2 and MDM4 to degrade p53, leading to an anti-apoptosis phenotype [44–46]. USP4 and USP5 inhibit p53 expression, but the molecular mechanism has yet to be elucidated [47–49]. USP10 can interact with G3BP2 to block p53 signaling and subsequently contributes to a poor prostate cancer prognosis [50]. However, in lung cancer, USP10 can inhibit cell growth and invasion by stabilizing PTEN, suggesting that the roles of USP10 in the different cancer types are distinct [51]. USP15 stabilizes MDM2 to regulate p53 and NFATc2 in cancer cells and T cells, respectively, resulting in tumor cell apoptosis and antitumor T cell responses [52]. USP24 can stabilize p53 but not c-Myc to inhibit tumorigenesis. USP42 was reported to stabilize TP53, but USP42 knockdown inhibits cancer formation, implying that other unknown factors related to cancer formation may exist [53]. USP2 stabilizes MDM2 and MDM4 to inhibit the Fas/p53 pathway during tumorigenesis [46, 54]. USP5 inhibits the p53 pathway [55]. USP7, USP10 and USP24 can stabilize p53 to inhibit cancer formation [45, 56, 57]. Our previous studies indicated that USP24 is downregulated in patients with early stage lung cancer. Overexpression of USP24 induces apoptosis by stabilizing securin and Bax, respectively [23]. USP27X stabilizes BCL2L11 to increase the anti-apoptotic effects of MAPK activity [58]. USP30 also participates in inhibiting apoptosis by stabilizing Parkin [59].

### The roles of USPs in cancer malignancy

Disrupted regulation of protein ubiquitination is a trigger of various diseases, including cancer. An increasing number of USPs have been shown to be involved in cancer malignancy. All USPs that are involved in cancer malignancy through the regulation of different pathways are then discussed.

#### USPs are involved in EMT and the stemness of cancer

USP11 stabilizes Snail to promote EMT in ovarian cancer [60]. USP24 also enhances TGFβ-induced EMT and metastasis of breast cancer [61]. Several previous studies have indicated that USP21 affects stem cells by stabilizing Nanog and IL8 [62]. Inhibition of USP34 induces EMT and stemness in mammary epithelial cells [63]. Previous reports indicated that USP47 promotes colorectal cancer EMT and malignancy by stabilizing Snail and activating the Wnt signaling pathway [64].

#### USPs regulate related pathways to control cancer metastasis

According to previous studies, several important cancer-related pathways are regulated by various USP members.

The JNKs-STATs compose an important pathway for cancer malignancy. Recent studies indicated that STAT3 activation represses USP7, leading to colon cancer development [65]. Another recent study indicated that USP3 mRNA functioned as a sponge for miR-224 to increase the level of SMAD4, resulting in colorectal cancer metastasis [66]. However, the role of USP3, as a deubiquitinase, is still not known [67]. CYLD controls c-Myc expression through a JNK-dependent signaling pathway in hepatocellular carcinoma [68].

The NFκB pathway is important for physiological and pathological progression, including inflammation and cancer progression. Many recent studies have shown that ubiquitination regulates not only protein degradation but also protein activity by modulating the interaction between proteins. Several USPs have been reported to be involved in the NFκB pathway [69]. USP6 is involved in the activation of the NFκB pathway, thus positively regulating tumorigenesis; however, the molecular mechanism is not yet known. USP11 can negatively regulate the NFκB pathway by stabilizing IκB [70]. USP18 inhibits the NFκB pathway by targeting TAK1 and NEMO for deubiquitination [71]. USP21 stabilizes IL33 to increase the signal transduction of NFκB [72]. Many studies have revealed that CYLD can inhibit NFκB signal transduction by regulating various factors, such as TRAF2/6, NEMO and Tak1 [73]. The polyubiquitination of TRAFs can increase the recruitment of other related proteins to induce the NFκB signaling pathway. USP4 and USP20 can promote the cell migration and invasion activities in breast cancer by inhibiting NFκB activation via deubiquitination of TRAF2 and TRAF6 [74]. Our recent study also indicated that USP24 can induce the NFκB pathway by stabilizing the βTrCP, which is the E3-ligase of IκB and DNMT1, causing the degradation of IκB and DNMT1 [75]. Regulation of USP35 by the miR let-7a can inhibit NFκB activation via deubiquitination and stabilization of ABIN-2 protein to inhibit cancer progression [76].

The TGFβ pathway is involved in several aspects of cancer progression, including cancer malignancy [77]. Different USPs regulate the TGFβ pathway by stabilizing different factors in this pathway [78]. USP4 and USP15 can stabilize TGFβ receptor type 1 to increase TGFβ-mediated EMT, leading to metastasis of hepatocellular carcinoma and glioblastoma [79–81]. A recent study indicated that a long noncoding RNA, H19, can compete with the binding of miR-148a to USP4 mRNA to increase the signaling activity of TGFβ [82]. USP9X can control the monoubiquitination of SMAD4 to regulate TGFβ-mediated cancer metastasis [83]. According to previous studies, USPs are crucial for the regulation of the TGFβ-mediated pathway [84].

The Wnt pathway is important for cancer EMT and metastasis [85]. USP4 can positively regulate the Wnt signaling in colorectal cancer [86]. Previous studies indicated that USP9X increases adhesion by destabilizing β-catenin [87]. USP14 and USP34 are required for Wnt signaling, but the detailed molecular mechanism is not yet known [88].

#### USPs are involved in the tumor-associated microenvironment

Our recent study found that USP24 is increased in M2 tumor-associated macrophages (TAMs), thereby promoting lung cancer malignancy through an increase in IL6 expression [75]. Increasing evidence indicates that TAMs are important for cancer malignancy and drug resistance [89–91]. Therefore, more USPs that are involved in regulating the tumor-associated microenvironment are expected to be identified in the future.

### The roles of USPs in DNA damage repair activity

DNA damage repair activity is related to the genomic integrity. A decrease in the DNA damage repair activity causes drug resistance under drug treatment, such as chemotherapy. According to recent studies, many deubiquitinases are involved in DNA damage repair pathways, indicating that deubiquitinases may be important for the induction of drug resistance. USP1 participates in restoring sensitivity to cisplatin in drug-resistance lung cancer cells by stabilizing FANCD2 [92]. USP3, 5 and 11 have been reported to be involved in increasing DNA damage repair activity by activating the DDR pathway [67, 93, 94]. USP8 may participate in TKI-induced drug resistance by increasing the levels of several receptor tyrosine kinases, including EGFR, ERBB2, ERBB3, and MET [95]. However, no substrate has been found to date. A recent study indicated that USP14 may be involved in cisplatin resistance by modulating the Akt/ERK signaling pathway in gastric cancer [96]. USP21 increases DNA repair and tumor growth by stabilizing BRCA2 [97]. USP22 promotes resistance to EGFR-TKIs by stabilizing EGFR in EGFR-mutant lung adenocarcinoma [98]. A recent study also indicated that the loss of USP22 causes to myeloid leukemia upon Kras activation through a PU.1-dependent mechanism [99]. USP22 induces cisplatin resistance in lung cancer by regulating γH2AX-mediated DNA damage repair and Ku70/Bax-mediated apoptosis [100]. USP22 knockdown increases the chemosensitivity of hepatocellular carcinoma cells to 5-FU by upregulating Smad4 and suppressing Akt [101]. USP26 is involved in the HR-dependent repair pathway. USP34 inhibits EMT and cancer stemness and may therefore induce more resistance to the drug treatment [102]. USP26 and USP37 participate in HR repair pathway by counteracting RAP80 [103]. USP47 promotes gastric cancer growth by regulating RelA. Many USPs discussed here are involved in DNA damage repair pathways, suggesting that USPs may be the potential targets for drug development of drug resistance in the future.

### USPs as targets for drug development in cancer prevention

In the past ten years, an increasing number of studies have indicated that most of USPs positively regulate cancer progression, including cell growth and malignancy. Recently, more inhibitors of USPs have been identified (Table 1). Most of the inhibitors can block more than one USP. Thus, increasing the specificity and effect of the inhibitors should be important in the future development. Herein we discussed how to develop a specific inhibitor of USPs. The development of USP inhibitors has resulted in a range of small molecule inhibitors and has been summarized in previous reviews [104, 105]. Many identified USP inhibitors have been suggested to have paninhibitory activity [104, 105]. For example, compound WP1130 has a broad panenzymatic DUB profile and can directly inhibit USP9x, USP5 and USP14 [106, 107]. However, this paninhibition may produce unwanted side effects. Designing a drug targeting a specific USP has proven challenging. This is due to the similarity of the conserved catalytic domain of the USP family. Therefore, identifying nonconserved regions is useful for designing specific USP inhibitors. In addition, further research on the interactions between compounds and the USP catalytic site is needed.

**Fig. 1:**
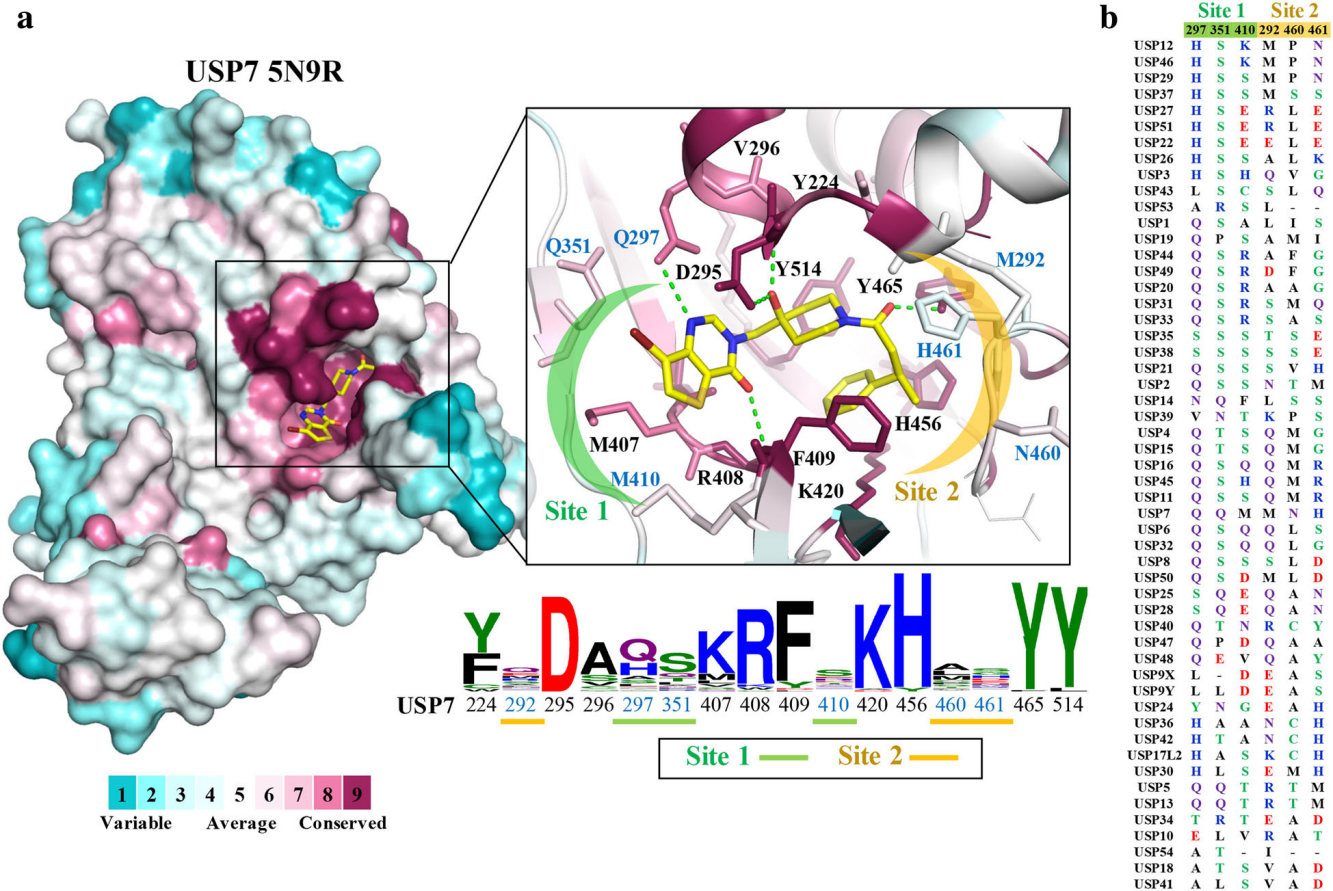
The USP family of proteins contain conserved and nonconserved catalytic regions. **a** Conservation score of the USP residue. The structure of USP7 (PDB ID: 5N9R) is used as a reference. Regions are shaded in blue, indicating nonconserved, or red, indicating conserved. The insert shows USP7 catalytic site residues and cocrystal ligand (yellow) as a stick model. The sequence pattern for the USP protein family is shown, with the USP7 sequence as a reference. Sites are highlighted as shown. The green line denotes the hydrogen bond. **b** Sequence alignment of Site 1 (green) and Site 2 (yellow). The sequence pattern figure was produced using the weblogo3 server (http://weblogo.threeplusone.com/create.cgi).

Sequence conservation analysis can provide clues for designing a selective inhibitor against a target protein. Using the crystal structure of a target protein, researchers can infer interactions in the catalytic domain to identify and design selective inhibitors. A sequence conservation analysis of USP was performed for this review. USP domain sequences were obtained from the UniProt Consortium [108]. A multiple sequence alignment (MSA) was performed using T-Coffee (http://tcoffee.crg.cat). Next, the MSA was submitted to the Consurf server (http://consurf.tau.ac.il/2016/) to identify conserved and nonconserved sequences. Each residue position was assigned a conservation score from variable (1) to conserved (9). Finally, the conservation score was mapped to the structure of USP7. Conserved and nonconserved regions exist in the USP catalytic domain (Fig. 1). For example, USP7 residue F409 has a high conservation score of 9. Residue F409, when USP7 is in complex with an inhibitor, adopts a conformation that produces a hydrophobic region that can be exploited by an inhibitor [109]. With the absence of crystal structures in complex with an inhibitor for other USP family members, analyzing the catalytic domain sequence remains crucial for designing possible inhibitors.

The sequence conservation analysis of the catalytic domain produced two nonconserved regions, designated Site 1 and Site 2. These are unique regions that vary between the USP family members and may be used to design a selective compound (Fig. 1a). The side chains of USP7 residues Q297 and Q351 are angled toward the Site 1 region. This allows possible hydrogen bond formation between a compound and USP7. However, the sequence analysis revealed different types of amino acids in these positions for USP family members. For instance, residue Q297 of USP7 is replaced by an alanine residue in USP18 and 54 (Fig. 1b). The alanine residue contains a shorter side chain than USP7 residue Q297. Furthermore, the alanine residue would not facilitate a hydrogen bond with its side chain. As a result, the catalytic region at Site 1 may be larger in other USP family members. This suggests that a compound with a larger nonpolar functional group would form additional van der Waals interactions with alanine. Such molecules may be more selective toward USP family members with alanine in this position. Many USP family members contain a serine at the 351 position (Fig. 1b). The serine side chain is shorter than the glutamine residue side chain. USP18 and USP41 contain an alanine and a threonine residue at the USP7 residue Q297 and Q351 position, respectively (Fig. 1b). This would suggest a larger Site 1 region. For example, the analysis suggests that USP18 and USP41 may have a larger Site 1 region. This region can accommodate a larger compound as well as a possible hydrogen bond with the threonine side chain to yield a selective USP18 or USP41 inhibitor. Finally, USP7 residue M410 occupies a region in the periphery of the USP7 catalytic site. Many USP family members contain residues at this position that are negatively charged. The presence of glutamate and aspartic acid residues at this position may form a salt bridge with a compound that has a positively charged functional group to make a specific interaction. Thus, the sequence conservation analysis suggests that a nonconserved pocket can be used to design selective USP inhibitors.

Site 2 is the other identified nonconserved region. This region consists of USP7 residues M292, N460 and H461 (Fig. 1a). According to the reference structure USP7, the side chains of residues at positions 292 and 460 face away from the catalytic region. This suggests that no direct interactions between compounds and the residue side chain occur with this region. However, the residue type at position 461 in USP7 is variable among the USP family (Fig. 1b). The side chain of residue USP7 H461 points inwards toward Site 2. This suggests that interactions at this position can greatly aid in USP selectivity. For example, USP12 contains an asparagine residue at this position and can form a hydrogen bond with a compound in this region. Possible hydrogen bond formation is also observed at this position with a serine residue in USP37. USP37 may also have a larger catalytic region at Site 2 due to the shorter side chain of serine. As a result, USP37 may be able to accommodate a compound with a larger moiety at Site 2. In total, the sequence conservation analysis identified two nonconserved sites. Interactions with the nonconserved sites present the possibility of designing a selective UPS inhibitor.

## Conclusion

Post-translational modification of protein is important for maintaining the physiological function. Dysregulation of protein ubiquitination will induce many diseases, such as cancer. E1/E2/E3-ligases and deubiquitinases regulate protein ubiquitination to control the function and stability of protein. Although many studies have addressed the importance of the USPs in cancer progression, several issues about USPs are still unknown. First, most of the substrates contain more than one deubiquitinases, why are more deubiquitinases needed to regulate the same protein? Second, according many previous studies, a lot of USPs are involved in the DNA damage repair activity, implying USPs may be related to drug resistance during cancer treatment. Therefore, more in-depth studies for clarifying the molecular mechanism are important. Finally, many USPs have been used as the target to develop the inhibitors. How to develop the inhibitors with more effective, low side effect and higher specificity is the most important issue in the future.
